# Ultra-high-resolution brain MRI at 0.55T: bSTAR and its application to magnetization transfer ratio imaging

**DOI:** 10.1016/j.zemedi.2024.12.001

**Published:** 2025-01-17

**Authors:** Grzegorz Bauman, Roya Afshari, Oliver Bieri

**Affiliations:** a Department of Biomedical Engineering, University of Basel, Allschwil, Switzerland; b Department of Radiology, Division of Radiological Physics, University Hospital Basel, Basel, Switzerland

**Keywords:** MRI, Balanced steady-state free precession, bSTAR, Magnetization transfer, Brain

## Abstract

**Purpose:**

This study aims to evaluate the feasibility of structural sub-millimeter isotropic brain MRI at 0.55 T using a 3D half-radial dual-echo balanced steady-state free precession sequence, termed bSTAR and to assess its potential for high-resolution magnetization transfer imaging.

**Methods:**

Phantom and in-vivo imaging of three healthy volunteers was performed on a low-field 0.55 T MR-system with isotropic bSTAR resolution settings of 0.87 × 0.87 × 0.87 mm^3^ and 0.69 × 0.69 × 0.69 mm^3^. Furthermore, off-resonance mapping was performed using 3D double-echo spoiled gradient imaging. For magnetization transfer (MT) MRI, the RF pulse duration of the 0.87 mm bSTAR scan was modified. Data were reconstructed using a GPU-accelerated compressed sensing algorithm. Magnetization transfer ratio (MTR) maps were calculated from two bSTAR scans with and without RF pulse prolongation. The MTR scan took 5 minutes and the reproducibility was assessed through repeated scans.

**Results:**

Off-resonance mapping revealed that bSSFP brain imaging with TR < 5ms is essentially free of off-resonance-related artifacts even near the nasal cavities. Phantom and in-vivo scans demonstrated the feasibility of sub-millimeter isotropic bSTAR imaging. MTR maps obtained with high isotropic resolution bSTAR showed contrast between white and gray matter in agreement with expectations from high-field studies. The MTR measurements were highly reproducible with an average inter-scan MTR peak value of 43.3 ± 0.3 percent units.

**Conclusions:**

This study demonstrated the potential of sub-millimeter and artifact-free morphologic brain imaging at 0.55 T using bSTAR leveraging the advantages of low-field MRI, such as reduced susceptibility artifacts and improved radio-frequency field homogeneity. Furthermore, MT-sensitized bSTAR brain MRI enabled whole-brain MTR assessment within clinically feasible times and with high reproducibility.

## 1 Introduction

Generally, mid to low-field MRI is challenged by low signal-to-noise and thus typically by limited spatial resolution [1], [2]. Only recently, however, balanced steady state free precession (bSSFP) has shown compelling results for high-resolution isotropic structural lung MRI [3]; providing sub-millimeter isotropic resolution at 0.55 T within clinically feasible scan-times [4]. Motivated by these results, ultra-high resolution bSSFP-based brain MRI might close some of the gaps between what can be achieved to what is desired or required for successful brain MRI at low as compared to high fields. Typically, however, its intrinsic and prominent T2/T1 contrast is not used for neurological MRI in the clinical setting but it was demonstrated that bSSFP is highly sensitive to magnetization transfer effects [5], [6].

Magnetization transfer (MT) was first demonstrated by Wolff and Balaban in the late 1980s, introducing a novel MR contrast beyond conventional T1, T2 and T2* weighted imaging [7]. Over the past decades, MT-based MRI methods have attracted considerable clinical interest to probe the source of various abnormalities in the brain, such as tumors [8], [9], Alzheimer’s disease [10], [11], [12], [13], multiple sclerosis [14], [15], [16], [17], [18], or to monitor brain development [19], [20], [21], [22].

The most commonly used method for MT imaging is based on spoiled gradient echo (SPGR) techniques. In this approach, additional radio-frequency (RF) pulses are used with a certain off-resonance and a high flip angle within every repetition time (TR) interval to induce a strong saturation of the macromolecular pool protons [23]. Upon magnetization exchange processes, the saturation of the macromolecular pool protons is transferred to mobile ones, leading to a prominent observable loss in the MRI signal, as compared to a scan without the extra MT-preparation RF pulses. It is evident, that the need for such repetitive MT-preparation modules considerably lowers the overall sequence acquisition efficiency. In contrast, no such MT-preparation modules are required for bSSFP, since MT effects can be modulated by a simple adaptation of the duration of the RF pulses used for excitation [6]. As a result, a high sequence efficiency can be maintained and it has been shown that 3D bSSFP-MT offers a three to four fold increase in the SNR for magnetization transfer ratio (MTR) imaging of the brain as compared to contemporary 2D SPGR-MT protocols at high field [24].

However, strong off-resonances can lead to distinct signal drops with bSSFP, so called banding artifacts that appear for alternating phase bSSFP for local phase accruals close to ±π within its repetition time. Successful bSSFP brain MRI may thus be hampered in regions of prominent susceptibility variations, such as near the nasal cavities. With decreasing main magnetic field, however, off-resonances drop and MT imaging with bSSFP becomes increasingly robust. Moreover, lowering the main magnetic field strength not only helps to reduce off-resonance-related artifacts but also provides a more favorable relaxation time ratio for bSSFP (i.e., an increased T2/T1 ratio and thus an increased SNR), as well as, a more homogenous radio-frequency excitation field.

In this work, we first explore the limits in TR and resolution for artifact-free bSSFP-based brain MRI using a half-radial dual-echo bSSFP pulse sequence (termed bSTAR [4]) in combination with an iterative compressed sensing reconstruction. In the second step, we examine the prospects of the developed high-resolution bSTAR protocol for whole brain MTR imaging.

## 2 Material and methods

MRI was performed on a commercially available 0.55 T low-field MR-system (MAGNETOM Free.Max, Siemens Healthineers, Erlangen, Germany) equipped with low-performance gradients (25 mT/m amplitude, 40 mT/m/ms slew rate). The 12-channel head coil was used for signal reception. Written informed consent was obtained from participants and measurements were approved by our local ethics committee. To assess the resolution limits of bSTAR an accredited American College of Radiology (ACR) structural phantom was used [25]. In-vivo examinations were performed in three healthy volunteers (two females and one male with average age of 31.3 years) using a product SPGR sequence for B0-mapping and a custom bSTAR sequence for MTR imaging.

### 2.1 Numerical simulations

Numerical simulations were performed in Matlab R2022a (The MathWorks, Inc., Natick, MA, USA). Simulations of the on-resonant bSSFP magnetization (*M*_xy_) as a function of the relaxation times (T1, T2), the TR and the flip (α) were performed using the standard signal equations; as stated in detail elsewhere [26]. Due to the higher T2/T1 and higher proton density (PD) of the aqueous resolution phantom as compared to brain tissue, the flip angle for the phantom bSTAR scan was adjusted (lowered) to yield approximately the same signal as brain white matter:

(1) $${\left\{PD\cdot M_{\mathrm{xy}}\left(T_{1},T_{2},\alpha \right)\right\}}_{\mathrm{phantom}}\approx {\left\{PD\cdot M_{\mathrm{xy}}\left(T_{1},T_{2},\alpha =40\;{}^{\circ}\right)\right\}}_{\mathrm{brain}\;white\;matter}$$

The T1 and T2 times of the resolution phantom were 150 ms and 100 ms. For brain white matter, a T2/T1 of 90 ms/450 ms and a proton density of 0.7 (relative to the aqueous resolution phantom) was assumed [27], [28], [29]. For TR << T2, flip angle settings are marginally dependent on the exact choice of the TR. An inspection of Eq. (1) yielded a flip angle of 18° for the phantom scan.

### 2.2 Phantom and in-vivo experiments

For off-resonance mapping of the brain, a single volumetric SPGR scan was performed with a predefined shim setting (i.e., in tune-up mode) using two echo times, TE(1) = 4 ms and TE(2) = 12 ms. The other sequence parameters were: TR of 17 ms, acquisition time (TA) of 2:23 min, flip angle of 15°, field-of-view of 224 × 256 × 192 mm^3^, acquisition matrix of 112 × 128 × 96, isotropic resolution of 2 × 2 × 2 mm^3^, and a bandwidth of 180 Hz/pixel.

MRI with bSTAR was performed with a non-selective RF excitation pulse and one single ADC spanned over the entire length of the bipolar readout gradient. The resulting two half-echoes were sampled with center-out and center-in radial projections following an Archimedean spiral trajectory. More technical details about the pulse sequence can be found in [3], [30].

To explore sub-millimeter resolution limits, we used two acquisition setups for the phantom scans: one with nominal high isotropic resolution (HR) of 0.87 × 0.87 × 0.87 mm^3^ (TR of 2.86 ms) and one with nominal ultra-high isotropic resolution (UHR) of 0.69 × 0.69 × 0.69 mm (TR of 3.92 ms). The theoretical SNR penalty of 0.5 for the 0.69 mm as compared to the 0.87 mm is accounted for by the increase in the overall scan time by about a factor of 5 (1:57 min against 9:45 min).

Generally, MT-weighting is reflected by a depletion of brain tissue signal and in the MTR calculation (c.f. Eq. (2)) noise is further increased. Thus, the HR setting is used for the generation of MTR contrast in the brain. As for bSSFP, MT effects with bSTAR were modulated by a simple adjustment of the RF pulse duration: a short RF pulse (and thus with short TR of 2.86 ms) for MT-weighting and a long RF pulse (and thus with long TR of 4.52 ms) for non-MT-weighting [6]. In addition, one in vivo brain UHR bSTAR scan was acquired, as for the phantom scans. A summary of all relevant bSTAR protocol settings is given in Table 1.

**Table 1:** Scan parameters for ultra-high-resolution (UHR) and high-resolution (HR) bSTAR brain imaging used in this work. MTR imaging was performed with HR bSTAR settings using RF pulse modulation. The MT-weighted scan was performed with short TRF whereas for the non-MT-weighted scan a long TRF was used.

**Sequence**	**UHR bSTAR**	**HR bSTAR (non-MT)**	**HR bSTAR (MT)**
FOV [mm]	256 × 256 × 256	256 × 256 × 256	
Nominal resolution (iso)[mm]	0.69	0.87	
Bandwidth [Hz/pixel]	631	775	
Samples per half-projection	240	208	
Number of spokes	150’000	40’000	
Number of echoes	2	2	
RF pulse duration (TRF) [µs]	600	1800	140
TE1 / TE2 [ms]	0.33 / 3.51	0.93 / 3.51	0.1 / 2.68
TR [ms]	3.92	4.52	2.86
Flip angle [ms]	40	40	
Acquisition time [min]	9:45	3:01	1:57
Undersampling factor (R)	3.1	8.0	

### 2.3 Data reconstructions and evaluations

Data from the bSTAR scans were reconstructed off-line to their nominal isotropic resolution using a GPU accelerated compressed sensing reconstruction with a fast iterative shrinkage-thresholding algorithm (FISTA) [31]. K-space trajectory corrections were performed using a measurement of the gradient input response function as described by Vannasjo et al. [32]. The data acquired at both echoes (i.e., obtained from the center-out and center-in half-radial projection) was reconstructed separately and added in the complex space after the reconstruction process. The reconstruction time using 12 FISTA iterations (regularization weight λ = 0.05) of a HR dataset with a reconstruction matrix 384^3^ took about 80 seconds and of UHR dataset with matrix of 512^3^ took about 6 minutes. The coil sensitivity profile obtained during the reconstruction of the non-MT HR dataset was exported and used for the reconstruction of the MT-weighted dataset to avoid a signal intensity bias. The in-house developed software for image reconstruction was written in C++ (GNU Compiler Collection 13.2 64-bit on Linux operating system) and CUDA Toolkit 12.3 (NVIDIA Corp., Santa Clara, CA, USA). The reconstruction workstation was equipped with 2x Epyc 7502 CPU (AMD Inc. Santa Clara, CA, USA) and Quadro RTX 8000 GPU (NVIDIA Corp.).

MTR contrast is reported in percent units ([pu]) and was calculated from the two, i.e., non-MT-weighted (S) and MT-weighted (S_MT_), bSTAR acquisitions, as usual [33]:

(2) $$\mathrm{MTR}=100\times (S-S_{\mathrm{MT}})/S$$

To assess the reproducibility of the MTR scan, pairs of MT-weighted and non-MT-weighted bSTAR scans were acquired twelve times. To this end, the volunteer was repositioned inside the MR-scanner prior to each consecutive set of MTR scan. Subsequently, an MTR peak histogram analysis was performed for each derived MTR contrast image. A box plot was generated for the retrieved MTR histogram peak positions, serving as indicator of the repeatability for the scan–rescan experiment.

For the scan-rescan experiment and for the off-resonance map, skull-stripping of the SPGR and of the bSTAR scans was performed using the standard software package FSL (FMRIB Software Library v6.0, Oxford, UK). No image registration was performed between the non-MT and the MT-weighted bSTAR scan used to calculate MTR maps.

## 3 Results

**Figure 1:**
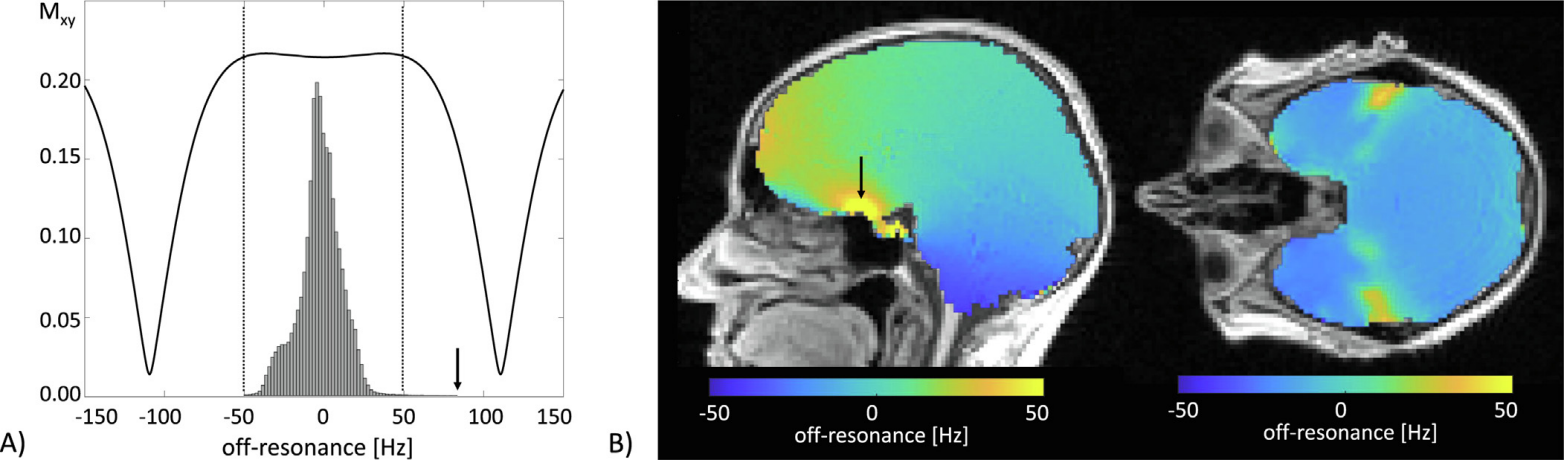
Analysis of off-resonances in human brain at 0.55T (volunteer #1) measured with a predefined shim setting (tune-up mode). (A) The bSSFP frequency response function for brain white matter (T2/T1 = 450 ms/90 ms) and a TR of 4.52 ms. A maximum off-resonance of about 80 Hz is observed above the nasal cavity (indicated by the arrow). For a typical TR of less than 5 ms, off-resonances fall within the pass-band region of bSSFP. (B) Sagittal and axial off-resonance maps showing the region around the observed peak off-resonances (black arrow). For 99.8% of brain voxels, off-resonances were between ±50 Hz.

An example histogram of the observed off-resonance frequencies for in vivo brain at 0.55 T is shown in Fig. 1A. Overall, 99.8% of brain tissue typically does not exceed off-resonances larger than about ±50 Hz. Observed peak off-resonances appear near the nasal cavity (cf. Fig. 1B) with maximum values of about 80 Hz. For ease of comparison, bSSFP’s frequency response function is overlaid to the observed off-resonance histogram (see Fig. 1A). For the maximum TR of 4.52 ms (see Table 1), banding appears at about ±110 Hz (±1/(2TR)) and the passband width is ±74 Hz (±1/(3TR)) [34]. As a result, bSSFP brain MRI with an upper TR limit of less than about 5 ms is essentially free of off-resonance related image artifacts and can be considered ‘on-resonant’ (the off-resonance for almost all brain tissue is less than ±50 Hz and thus lies within the pass-band region of bSSFP).

**Figure 2:**
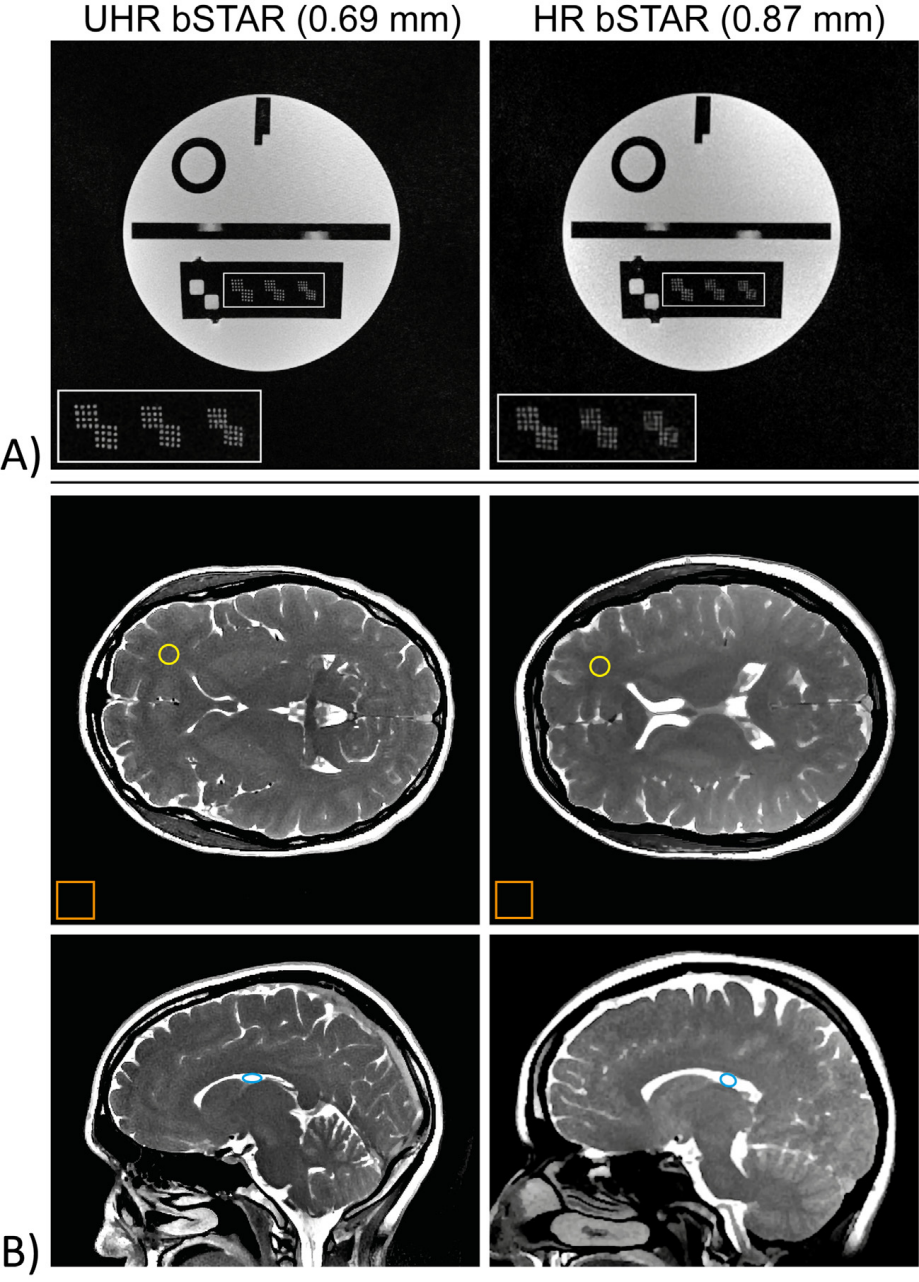
Ultra-high-resolution (UHR) and high-resolution (HR) bSTAR images of a resolution phantom (A) as well as axial and sagittal images obtained in volunteer #2 (left column) and volunteer #3 (right column) (B). The flip angle for the bSTAR phantom scan was adjusted (i.e., lowered) to match the expected signal of brain white matter (see Materials and Methods section). The resolution rods in the phantom has been marked with a white rectangle and zoomed in the left bottom corner for an improved visualization. The dimensions of the resolution rods (from left to right): 0.9 mm, 0.8 mm and 0.7 mm. Regions of interest placed on in-vivo images were used for calculation of the apparent SNR in the white matter (yellow circles), cerebrospinal fluid (blue circles) and in a noise-only region (orange rectangle).

Isotropic sub-millimeter bSTAR scanning is first assessed using a resolution phantom (see Fig. 2A). Note that the flip angle for the bSTAR phantom scan was lowered about two-fold as compared to the flip angle setting for the in-vivo brain scans, and thus imaging was performed with lowered SNR, in order to match the signal for the phantom scan to the signal of brain white matter (see Materials section). The sub-millimeter resolution rods (0.9 mm, 0.8 mm and 0.7 mm from left to right) in the lower-middle part of the phantom can be easily distinguished on the UHR acquisition (undersampling factor of 3.1), while the largest 0.9 mm resolution rods can be separately recognized on the HR scan (undersampling factor of 8.0). Moreover, no undersampling-related image artefacts (streaking artefacts) can be visually identified in the images. As a result, the phantom scans confirm the stated nominal sub-millimeter resolutions for the suggested protocol settings (see Table 1).

Corresponding example UHR and HR MT-weighted bSTAR brain scans are shown in axial and sagittal orientation in Fig. 2B. As for the phantom images, no undersampling artefacts can be identified and the brain tissue is essentially free of any banding artifacts; even in regions near the sinuses, where one would expect the most distinct artifacts. Overall, from the adaptation of the UHR scan time, both UHR and HR in vivo brain scans result in a similar apparent SNR (see Table 2). It must be noted, however, that the reported apparent SNR values allow only a comparison between images generated using the same reconstruction algorithm.

**Table 2:** Apparent SNR values measured in the white matter and cerebrospinal fluid in the UHR bSTAR, HR bSTAR (non-MT) and HR bSTAR (MT) acquisitions. For the definition of regions of interest (ROI) used for calculation of the SNR; cf. Fig. 2.

	UHR bSTAR	HR bSTAR (non-MT)	HR bSTAR (MT)
White matter	20.6 ± 1.5	39.8 ± 1.9	21.4 ± 1.6
Cerebrospinal fluid	68.1 ± 2.2	84.4 ± 2.8	76.8 ± 4.3

**Figure 3:**
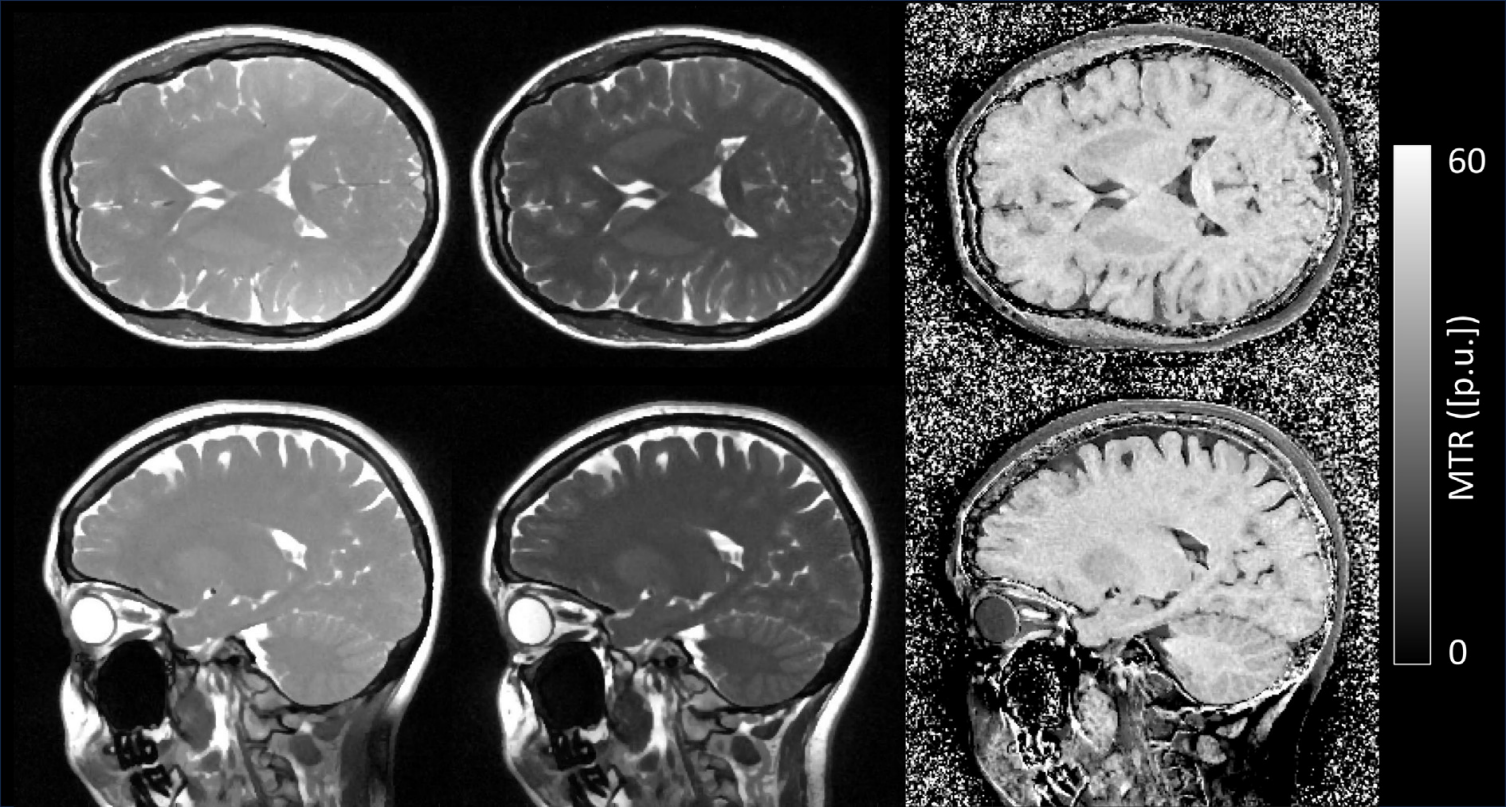
MTR brain imaging using bSTAR. Example slices obtained in volunteer #2 are shown in axial and sagittal orientations for non-MT-weighted (left column) and MT-weighted (middle column) bSTAR MRI with corresponding MTR image (right column).

Example slices in axial and sagittal orthogonal directions for non-MT-weighted and MT-weighted bSTAR MRI are shown in Fig. 3. An increase in the RF pulse duration (corresponding to a decrease in the RF pulse power) leads to a pronounced signal gain in brain tissue due to MT effects (and thus also of the apparent SNR, see Table 2), as reflected by the corresponding MTR contrast images. In addition, the axially reformatted MTR data has been animated in the Supplementary Material Video 1. At 0.55 T and for bSSFP, MTR values are highest for brain white matter, revealing a slight contrast between gray and white matter tissue; in agreement to what is observed at high field [6].

**Figure 4:**
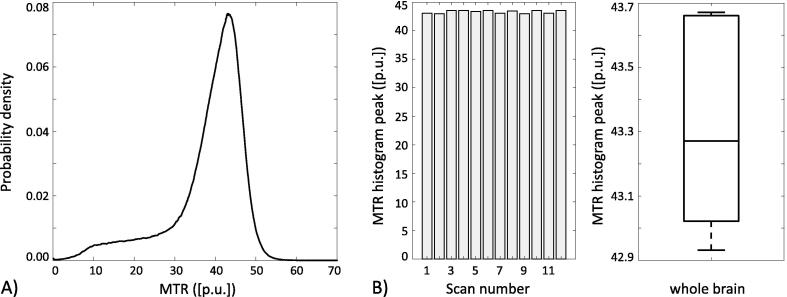
MTR imaging at 0.55T with MT-sensitized bSTAR: (A) Example MTR histogram for whole brain measured in volunteer #2. (B) Repeatability assessment from 12 scan–rescan experiments based on an MTR histogram analysis, showing the MTR peak value together with a box plot representing the median, lower and upper quartiles, as well as the maximum and minimum peak values.

An example of a whole brain MTR histogram (including CSF) derived using MT-sensitized bSTAR is shown in Fig. 4A. For the suggested protocol, brain tissue MTR values peak at around 40–45 pu. Moreover, MTR measurements were highly reproducible, showing an average inter-scan MTR peak value of 43.3 ± 0.3 pu (see Fig. 4B).

## 4 Discussion

At 0.55 T, bSTAR has demonstrated compelling results for imaging the lung; especially in terms of resolution [4]. Inspired by these results, the feasibility of ultra-high and high-resolution isotropic bSSFP-based brain MRI is evaluated in this work. As for the lung, bSTAR brain MRI is essentially free of any off-resonance related image distortions for standard TR settings.

Generally, in brain tissue, the contrast of bSSFP (and thus of bSTAR) is dominated by fluids and thus shows an apparent contrast similar to spin-echo (SE) T2-weighted MRI of the brain (e.g., see Fig. 2); especially if no off-resonance related image artifacts are present (local banding). In contrast to 3D SE imaging, however, bSTAR offers rapid high-resolution isotropic volumetric scanning. One of the current limitations of the UHR bSTAR brain protocol is its relatively long acquisition time of over 9 minutes. Recent deep learning denoising and reconstruction methods, however, have revealed unprecedented power to further enhance not only image quality but also to accelerate the acquisition [35], [36], [37]. Thus – although currently limited by rather extensive scan time requirements – UHR bSTAR brain MRI in combination with deep learning may yield a considerable boost in scan times. Future studies may thus focus on the evaluation of the clinical usability of bSSFP’s contrast as a possible surrogate for rapid whole brain isotropic “T2-like” weighted brain MRI.

Nevertheless, rapid HR isotropic brain MRI was shown to be feasible at low field with sub-millimeter resolution without any deep learning based denoising but using a state-of-the art compressed sensing algorithm for image reconstruction [31]. This setting was evaluated for rapid whole brain MTR imaging from two HR bSTAR brain scans with a suggested total scan time of only 5 minutes. The observed MTR contrast at 0.55 T agreed well with expectations from higher field strength, was highly reproducible and was essentially free of any off-resonance related image degradations. It is noteworthy, that sub-millimeter MTR MRI is likewise challenging at high field [24].

In contrast to MT-prepared SPGR, MT-sensitized bSSFP is, in general, much more efficient. Notably, in combination with half-radial encodings, such as with bSTAR, bSSFP sequence efficiency is especially high [3], [4]. Moreover, it has already been shown that under the constraint of identical SNR and scan time, Cartesian bSSFP offers a 3.4 times higher voxel size than MT-prepared SPGR at 1.5T [24]. Although in this study no direct comparison of MT-sensitized bSTAR with half-radial or radial MT-prepared SPGR was performed, similar results can be expected at 0.55T as compared to 1.5T. At high field, however, reliable high-resolution whole brain MTR imaging with MT-sensitized bSSFP can thus be hampered by the presence of prominent off-resonances, such as near the nasal cavities [38]. This possible issue was completely mitigated at low field, although we used a low-performance 0.55 T MR-scanner with reduced slew rate and gradient amplitudes, and thus with limited minimal TR settings, as compared to high-end clinical MR-scanners. Moreover, MTR contrast can be affected by transmit field inhomogeneity affecting reproducibility [39]. Although bSSFP-based MTR imaging is rather insensitive to excitation field inhomogeneities for optimal flip angle settings [6], this effect becomes mitigated not only for bSSFP but also for conventional MTR methods, such as SPGR.

## 5 Conclusions

In this study, we showed the feasibility of sub-millimeter and artifact-free morphologic brain imaging at 0.55T using bSTAR MRI. Furthermore, we demonstrated the potential of MT-sensitized bSTAR brain MRI for whole brain MTR assessment within clinically feasible times and with high reproducibility. Despite lower SNR, the advantages of low-field MRI including reduced sensitivity to magnetic field inhomogeneity, lower SAR and improved transmit field homogeneity, are especially appealing for bSSFP-based sequences and allows for high-resolution imaging when combined with an efficient acquisition scheme, such as offered by bSTAR.

## CRediT authorship contribution statement

**Grzegorz Bauman:** Writing – review & editing, Writing – original draft, Visualization, Software, Methodology, Investigation, Data curation, Conceptualization. **Roya Afshari:** Writing – review & editing, Writing – original draft, Visualization, Validation, Investigation, Formal analysis, Data curation. **Oliver Bieri:** Writing – review & editing, Writing – original draft, Visualization, Supervision, Resources, Project administration, Methodology, Investigation, Funding acquisition, Formal analysis, Conceptualization.

## Declaration of competing interest

The authors declare that they have no known competing financial interests or personal relationships that could have appeared to influence the work reported in this paper.

## Appendix A Supplementary data

Supplementary video 1 Animation showing axial view of a 3D MTR volume acquired in a healthy volunteer.
